# Cancer stem cells-driven tumor growth and immune escape: the Janus face of neurotrophins

**DOI:** 10.18632/aging.102499

**Published:** 2019-12-07

**Authors:** Viviana Triaca, Valentina Carito, Elena Fico, Pamela Rosso, Marco Fiore, Massimo Ralli, Alessandro Lambiase, Antonio Greco, Paola Tirassa

**Affiliations:** 1 Institute of Biochemistry and Cell Biology, National Research Council (CNR), International Campus A. Buzzati-Traverso, Monterotondo Scalo, Rome, Italy; 2 Institute of Biochemistry and Cell Biology, National Research Council (CNR), at Department of Sense Organs, University of Rome La Sapienza, Rome, Italy; 3 Department of Sense Organs, University of Rome La Sapienza, Rome, Italy

**Keywords:** cancer stem cells, NGF, immunesurveillance, tumor microenvironment, cancer innervation

## Abstract

Cancer Stem Cells (CSCs) are self-renewing cancer cells responsible for expansion of the malignant mass in a dynamic process shaping the tumor microenvironment. CSCs may hijack the host immune surveillance resulting in typically aggressive tumors with poor prognosis.

In this review, we focus on neurotrophic control of cellular substrates and molecular mechanisms involved in CSC-driven tumor growth as well as in host immune surveillance. Neurotrophins have been demonstrated to be key tumor promoting signaling platforms. Particularly, Nerve Growth Factor (NGF) and its specific receptor Tropomyosin related kinase A (TrkA) have been implicated in initiation and progression of many aggressive cancers. On the other hand, an active NGF pathway has been recently proven to be critical to oncogenic inflammation control and in promoting immune response against cancer, pinpointing possible pro-tumoral effects of NGF/TrkA-inhibitory therapy.

A better understanding of the molecular mechanisms involved in the control of tumor growth/immunoediting is essential to identify new predictive and prognostic intervention and to design more effective therapies. Fine and timely modulation of CSCs-driven tumor growth and of peripheral lymph nodes activation by the immune system will possibly open the way to precision medicine in neurotrophic therapy and improve patient’s prognosis in both TrkA- dependent and independent cancers.

## Introduction

### Cancer stem cells niche

Tumor tissues consist of heterogeneous cancer cells, including stem-cell-like subsets of cancer stem cells (CSCs), characterized by self-renewal and long-term clonal maintenance [1]. Thus, CSCs are not only responsible for initiating the tumor process, but they have long-term repopulation capacity in recurrent tumors. Also, CSCs show significant DNA repair capability and resistance to current chemo, radio, and immune therapies [2].

Based on the revised CSCs model, several genetically different subclones of CSCs may co-exist and expand according to their own hierarchy within the tumor bulk, and contribute to overall cancer heterogeneity [3]. The first evidence for CSCs came from the observation of a small subset of clonogenic cancer cells in acute myeloblastic leukaemia (AML) [4]. CSCs were subsequently identified in other tumor types, including multiple myeloma, breast, brain, prostate, and lung cancers. CSCs are tumorigenic even when transplanted in low numbers in experimental models [5,6]. They carry the neural stem cells marker CD133, which was identified first in human brain tumors, and then in many other solid tumors, like prostate, colon, and pancreas tumors [7].

Tumor aggressiveness is strictly linked to a process known as Epithelial-Mesenchymal Transition (EMT). Subpopulations of tumor cells that undergo EMT [8] induce apoptosis of neighboring non-cancerogenic endothelial cells [9] and disrupt epithelial junctions, becoming metastatic as Circulating Tumor Cells (CTCs). Above described events have been found to be common in distinct types of carcinoma, like head and neck cancer, esophageal cancer, breast cancer, lung cancer and melanoma.

In hematological malignancies the disease reinitiates from microscopic residual tumor, known as minimal residual disease (MRD), routinely analyzed through a technique called “liquid biopsy” [10]. Maintenance of undifferentiated and self-renewing CSCs relies on the “stem cell niche”, a microenvironment mainly represented by dendritic cells (DCs), tumor associated macrophages (TAMs), fibroblasts, tumor-specific T cells, and neutrophils [11,12]. The niche is enriched in factors promoting CSCs self-renewal, angiogenesis, tumor invasion and metastasis, as reviewed in [13,14]. To avoid tumor relapse and improve patient prognosis, several molecular targets and biomarkers have been suggested. The nuclear factor-kB (NF-kB), Notch, and phosphatidylinositol 3-kinase/AKT/mammalian Target of Rapamycin (PI3K/AKT/mTOR) pathways are the most relevant signaling platforms targeted for their involvement in CSCs metabolism, survival, proliferation, growth, invasion, and resistance to therapy [15,16]. Notch pathway is also involved in the immune surveillance process, promotes M1 macrophage polarization [17] and CD8+ T cells activation, and acts as a tumor-suppressor [18]. Furthermore, activation of the Interleukin-6 (IL-6)/ Signal transducer and activator of transcription 3 *(*STAT3)/Aldehyde Dehydrogenase 1 (ALDH1) pathway by adipose tissue-derived vesicles, cytokines, and circulating factors has been recently implicated in tumor stemness and aggressiveness, particularly in breast cancer [19–21].

Several inhibitors preferentially targeting CSCs have been tested *in vitro* and in preclinical studies, like the pan-PI3K inhibitor B591 [22] and the dual PI3K/mTOR inhibitor VS-5584 [23]. However, novel therapies are still demanding, because of the limited efficacy and side effects of currently available CSCs-based targeting approaches.

Nowadays, immunotherapy represents the latest frontier of CSCs-based cancer therapy due to its broader range application over different cancer types. Here below, we will focus on the role of immune system attempted control against cancer growth and spreading, highlighting the double-edged sword of neurotrophins in cancer immunity and inflammation, of interest for the design of novel and efficient therapies targeting CSCs-driven tumors and metastasis.

### CSCs and tumor immune surveillance

### The immune surveillance hypothesis

The immune surveillance hypothesis states that the immune control of cellular homeostasis is the first line of host defense against carcinogenesis. The host immune system-tumor interplay consists of three essential phases: elimination, equilibrium and escape (reviewed in [24,25]).

*Elimination.* Exposure of immunogenic antigens by mutated or dying cells activates Natural Killer (NK) receptors NKGD and promotes proliferation of infiltrating CD8^+^ T cells by induction of major histocompatibility complex (MHC) class Ia, resulting in their clearance. In particular, a subset of high Interferon -γ (IFN-γ) secreting NK cells is at the forefront of innate response against cancer and it is responsible for Tumor Necrosis Factor (TNF)-related apoptosis-inducing ligand (TRAIL)-dependent lysis of tumor cells in mice [26]. Stress or necrosis induced signals, like Danger Associated Molecular Patterns (DAMP), are crucial for stimulating Pattern recognition receptor (PRR), like Toll-like receptor (TLR) and Nod-like receptor (NLR), elective effectors of innate immunity. *Equilibrium.* Premalignant stem cells are maintained in equilibrium with the adaptive immune response, which selects low-dividing and immune tolerant emerging subclones in a process called immunoediting Tumor stem cells are still dependent upon their niche and cancer metastasis is unlike to occur. *Escape.* The immune escape mainly relies on immune system aging and expansion of less immunogenic (immuneselection) and/or less immunosuppressive (immunesubversion) CSCs subclones (reviewed in [25]), resulting in overt tumors.

### CSCs driven immuneselection and immunesubversion

CSCs may escape the active clearance by hiding themselves to the immune system via the downregulation or lack of MHC class I (MHC-I) molecules, as observed in melanoma, prostate cancer, bladder, and colorectal carcinoma (CRC). In particular, CSCs undergo a switch in the MHC-I expression, reducing immune-activator MHC class Ia (HLA A-C) in favor of immune-inhibitory MHC class Ib (HLA E-G) molecules, and suppressing MHC class II (MHC-II) and costimulatory molecules, like CD40, B7-1 and B7-2. Moreover, CSCs lack the expression of ligand for activator NK receptors (NKp44, NKp30, NKp46 and CD16) and in turn upregulate ligands for inhibitor NK receptors (HLA-G), resulting in innate immunity repression.

Overall, immune escaping CSCs subclones hijack the host immune system response. They are able to 1) reduce the expression of M1 macrophages inhibitors CD200 and CD44 blocking macrophage M2 polarization and phagocytic activity, 2) produce several cytokines in the TME, like Transforming Growth Factor β (TGF-β), IL-4, IL-6, IL-10, paralyzing the immune system responses, 3) convert a subset of immature myeloid DCs into TGF-β-secreting cells, thus driving expansion of immunosuppressive regulatory T cells (Tregs) in lymphoid organs of tumor bearing mice [27,28], and 4) attract Tregs and Myeloid-Derived Stem Cells (MDSC), facilitating CSCs spreading and metastatization [29]. Further, mutations promoting CSCs survival outside the CSCs niche favor CSCs spreading and cancer metastasis. Tumor variants emerging after lymphocyte and cytokines selection are the first cause of mortality, because of their resistance to both chemo/radiotherapies and adoptive cell therapies.

### Immunotherapy

Accumulating results indicate that CSCs may develop resistance to standard cancer therapies, including chemo-radiotherapy and molecular targeted therapy, making more difficult to fight cancer with available clinical approaches. A recently adopted treatment is immunotherapy, stimulating the immune system surveillance against the tumor, and combining monoclonal antibodies, immune response modifiers, and vaccines. Unlike conventional chemotherapy resulting in secondary resistance, the co-inhibitory immune checkpoints (ICI) therapy revealed a significant long-lasting clinical effect in melanoma, non-small cell lung cancer, renal and bladder cancers, HNSC, CRC, and Hodgkin lymphoma [30–32]. ICI therapy with monoclonal antibodies anti-PD-1 and anti-Cytotoxic T-Lymphocyte Antigen 4 (anti-CTLA-4) promoted T cells migration and intratumoral invasion, thus supporting effective tumor elimination. Nowadays, the design of novel therapies facilitating tumor immunoediting by immune cells and/or exposing CSCs to the host immune response represent promising prospective treatments in solid tumor therapy.

### CSCs in oncogenic and therapeutic inflammation

The induction of chronic oncogenic inflammation by CSCs favors the release of pro-tumorigenic chemokines by innate immune cells, thus resulting in tumor cell growth, survival, and angiogenesis [33–35]. Indeed, the tumor itself has the chance to promote cancer metastasis taking advantage of oncogenic inflammation. The same chemokines, like INF-γ or TGF-β, seem to be involved in both immune surveillance and pro-oncogenic inflammation. Indeed, tumor promoting inflammation and tumor-suppressive immunity co-exist, rendering it more difficult to distinguish two phenomena with common patterns of immune cells and cytokines. A further level of complexity is introduced by therapeutic inflammation induced by chemotherapic drugs and radiotherapy. It subserves the effective eradication of the tumor mass by helping antigen-immune system cross-talk [24]. The deeper comprehension of the inflammasome regulation in CSCs metabolism and cancer might improve efficacy of molecular and cellular targeting in current therapy.

## Neurotrophins in CSCs-driven tumor growth

### Neurotrophins and neurotrophins receptors expression in cancer

**Figure 1:**
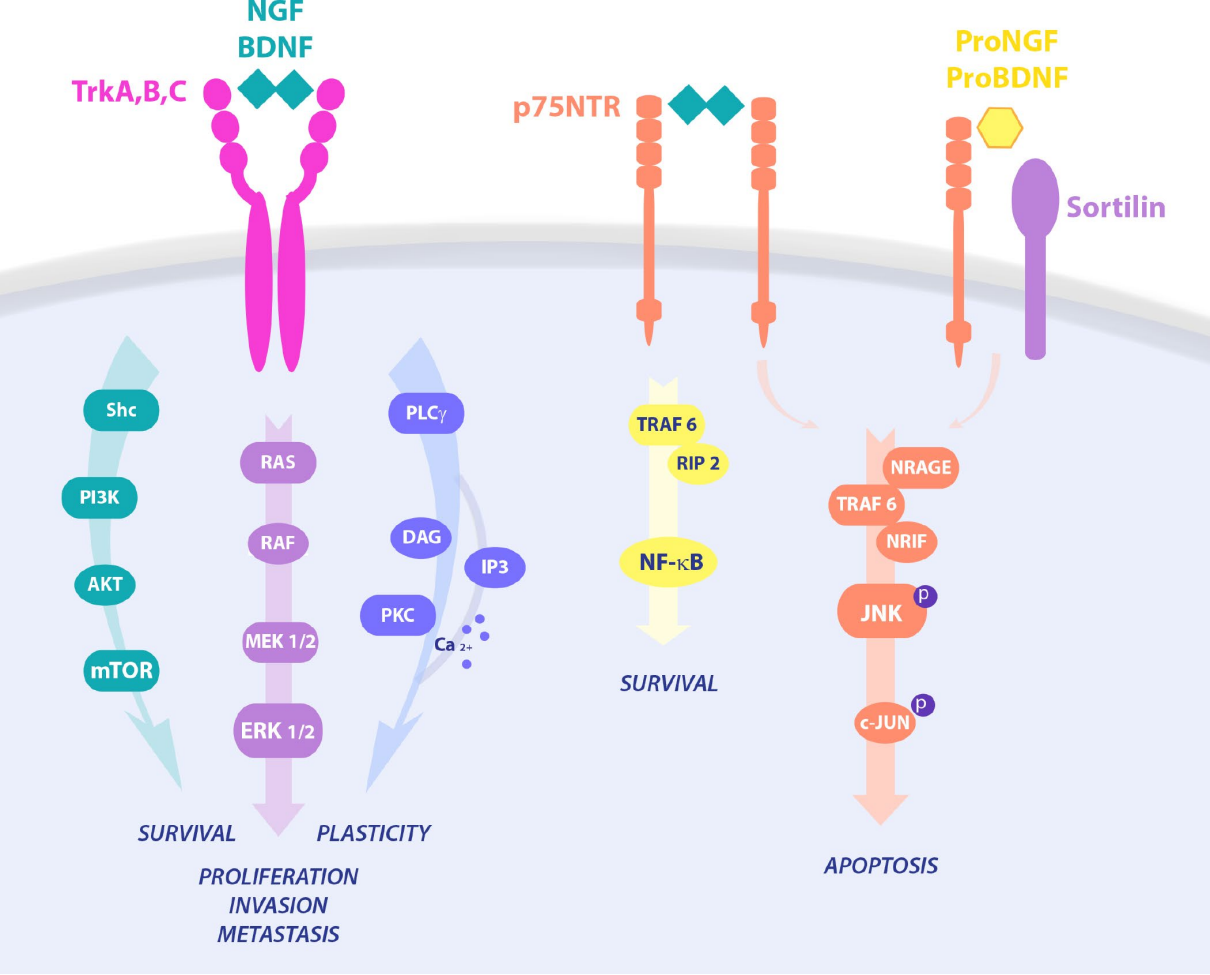
**Neurotrophins signaling pathways in cell survival and death.** NGF binds TrkA and p75 in a trimeric complex and mediates proliferation, differentiation, and survival via activation of different pathways, like PI3K/AKT, Ras/MAPK and PLC-γ. Upon p75NTR homo-dimerization, NGF is also able to activate NF-κB or JNK, resulting in RIP2 and NRAGE/NRIF signalings, respectively. On the contrary, proneurotrophins, and proNGF in particular, complex with p75NTR and sortilin, leading to activation of pro-apoptotic pathways and cell death.

Neurotrophic signaling has been strongly implicated in cell survival, proliferation and apoptosis (Figure 1). Unbalanced expression of neurotrophins Nerve Growth Factor (NGF), Brain-Derived Neurotrophic Factor (BDNF), Neurotrophin3 (NT3), and/or their receptors Tropomyosin related kinase A (TrkA), Tropomyosin receptor kinase B (TrkB), Tropomyosin receptor kinase C (TrkC), and common Neurotrophin Receptor p75 (p75NTR) have been reported in cancer [36,37]. Increased evidence pinpoints a central role of the neurotrophic pathways in cancer growth and progression. In particular, NGF and BDNF are considered diagnostic biomarkers for hepatic cancer (HC) and CRC. NGF level is 57.3 times higher in CRC than in normal colon tissue [38] and significantly correlates with esophageal squamous cell carcinoma (ESCC) and CRC growth and metastasis [39]. Moreover, NGF overexpression is sufficient *per se* to induce gastric cancer (GC) in rodent animal models [40]. In line with this, anti-NGF based therapies have been demonstrated to be a promising approach in tumor treatment, as well as in tumor associated cancer pain [41]. Further, inhibition of TrkA blocked tumor growth and reinforced chemotherapy in pancreatic ductal adenocarcinoma (PDAC) [39].

TrkA, TrkB, and TrkC proteins derive from *NTRK1, NTRK2* and *NTRK3* genes, respectively. Genetic aberrations of the *NTRK* genes, like point mutations, gene fusions, constitutively active splice variants are the most well validated oncogenic events in both infantile and adult cancers [42–44]. *NTRK1* genetic variants occur in tumors of neuronal type (neuroblastoma and medulloblastoma), but also in non-neuronal cancers, like thyroid, breast, lung, prostate, ESCC, PDAC, GC, HC, and CRC (reviewed in [39,45–48]).

Constitutively active TrkA isoforms generated by alternative splicing rearrangement, like TrkAIII (spliced exons 6, 7 and 9), lack the extracellular signal for membrane localization, induce sustained PI3K/AKT/NF-kB signaling, and cause DNA instability [49]. Oncogenic TrkA fusion products [50] potentiate NGF-dependent carcinogenesis in CRC, thyroid cancer, and AML, leading to the concept of *NTRK1* functioning as an oncogene [39]. Treatment of cancers presenting TrkA fusion proteins with selective Trk inhibitors resulted in a better prognosis [51,52]. Further, over-expression of the NGF specific receptor TrkA is considered a reliable index of tumorigenicity, invasiveness, and chemotherapy resistance in several types of squamous cancers [53].

*NTRK2* variants have been observed in glioblastoma and HNSC [54]. Also, TrkB overexpression has been observed in ESCC and GC, associated to anoikosis caused by decreased E-cadherin in GC, and to higher chemoresistance in ESCC, being thus considered a metastasis predictor and a strong indicator of bad prognosis [55].

On the contrary, TrkC is a conditional tumor suppressor acting as a dependence receptor and able to induce the caspase cascade in absence of its ligand NT3. NTRK3 is a conditional tumor suppressor epigenetically or genetically downregulated in CRC [56]. Thus, CRC cells characterized by the loss/dysfunctional mutation in the *NTRK3* gene acquire a selective advantage and contribute to CRC expansion.

As for the common neurotrophin receptor, p75NTR belongs to the tumor necrosis factor family of receptors. The expression studies on p75NTR led to conflicting results, with p75NTR demonstrated to be a tumor suppressor and a good prognostic factor in digestive cancers or a valuable index of tumor aggressiveness in ESCC and prostatic cancers [39,57–59]. In fact, p75NTR expression is negatively regulated and sometimes null in HC and GC as compared to normal mucosa [60], while its re-activation induces apoptosis through cell cycle arrest, as observed *in vitro* in HC cells [61]. Moreover, p75NTR is specifically overexpressed in prostate cancer cells, where its level correlates with high-risk prostate tumors with a poor prognosis (Gleason score >7), but not in normal and benign hyperplastic prostate epithelial cells [62]. On the other hand, p75NTR is a marker of chemo-resistant CSCs population in ESCC [59].

### Neurotrophins signaling pathway promoting CSCs survival/proliferation

Neurotrophins are regulators of developmental neuronal survival, growth and differentiation and mediate higher-order functions, like synaptic plasticity, learning, memory and behavior in adulthood, after injury, and in age-related neurodegeneration. NGF, BDNF, NT-3, and NT-4/5 are members of the neurotrophin family of growth factors [63,64]. Neurotrophins have preferential binding for specific receptors of the Trk family of receptor tyrosine kinases: NGF binds to TrkA, BDNF and NT-4 to TrkB, and NT-3 to TrkC. All neurotrophins are able to bind the common p75NTR. Further, the association of p75NTR with Trk receptors stabilizes Trk binding to its neurotrophin. Neurotrophin signaling includes Ras, PI3K, Phospholipase C-γ (PLC-γ), and mitogen-activated protein kinase (MAPK) activation leading to survival, proliferation and/or differentiation of target cells. Opposite, activation of p75NTR stimulates NF-κB and c-Jun N-terminal kinase (JNK), promoting inflammation and apoptosis, respectively [65]. In many cancers TrkA activates prosurvival downstream pathways upon NGF binding, while p75NTR binding to its preferred ligand proNGF and co-interactors, like sortilin, instructs pro-apoptotic signaling leading to cell death [66]. In particular, proNGF/p75NTR activates cell death in prostate cells, while their loss in prostate cancer allows tumor expansion and spreading [36]. However, ligand neurotrophins also show ambiguous behavior in carcinogenesis. In line, proNGF binds TrkA and sortilin in breast cancer, inducing the Sex Determining Region Y-box 2 (Sox2) and conferring higher invasiveness to CSCs in a p75NTR-independent manner [67]. Of note, both precursor and mature neurotrophins have been reported to promote tumor growth in breast cancer [68], where p75NTR has been also associated to a pro-survival effect [69].

NGF, BDNF and NT3 pathways represent survival and proliferation signals in CSCs, by activating the Son of Sevenless (Sos)-Ras-MAPK and Fibroblast growth factor receptor substrate 2 (Frs2)/Ankyrin Repeat-Rich Membrane Spanning (ARMS)-Crk pathways, leading to cAMP response element-binding protein (CREB) and NF-kB stimulation, and finally controlling key cellular check-points implicated in CSCs proliferation in glioma, HNSC, melanoma, and breast cancer [37]. BDNF/TrkB and NT3/TrkC signaling complexes have been shown to promote CSCs survival via AKT and Extracellular signal–Regulated Kinases (ERK) pathways in glioma [70]. Further, TrkB deletion in CSCs prevents tumor reappearance in recurrent triple negative breast cancer [71].

NGF survival signaling through the TrkA pathway follows three main routes of intracellular second messengers: 1) Src homology 2 domain containing (Shc)/PI3K/AKT leading to survival of breast and prostate cancers, 2) the Ras/MAPK induced proliferation and invasion in breast and prostate cancer, and cell death by autophagy in medulloblastoma and glioblastoma, 3) PLC-γ/PKC signaling involved in metastasis [36]. Interestingly, signaling via p75NTR strongly depends on binding to its interactors sortilin, Leucine Rich Repeat and Ig Domain Containing 1 (LINGO1), and Neurite outgrowth inhibitor (NOGO), switching from survival to cell death pathways, growth regulation and macrophage clearance, respectively. Indeed, p75NTR via Tumor necrosis factor receptor type 1-associated DEATH domain-dependent (TRADD-dependent), NF-kB and Brain expressed X-linked (BEX) drives a prosurvival effect in breast cancer and schwannoma, while being anti-cancerogenic via JNK-mediated apoptosis in prostate cancer cells and neurons [72–74]. Constitutive active TrkAIII variant induces an undifferentiated stem-like phenotype through increased expression of stemness genes like Nanog, Nestin, Sox2 and CD117, leading to the formation of larger neurospheres in SH-SY5Y neuroblastoma cell line [75]. The NGF-TrkA pathway induces p75NTR proteolytic processing and the release of the soluble p75NTR intracellular domain (ICD), which is central to AKT signaling and CSCs sustained proliferation in several tumor types [76,77], suggesting that the generation of the ICD domain is crucial for the NGF/TrkA pro-oncogenic pathway [39]. Of note, Rho GTPase-mediated recruitment of CD44 to the cell membrane by NGF-TrkA further contributes to CSCs stemness maintenance and survival, resulting in higher tumor aggressiveness [78]. According to this, pharmacological TrkA inhibition prevents p75NTR shedding and the proliferative effect of NGF observed in CSCs. Interestingly, the p75NTR has been observed in the mitotically quiescent CSCs population of the basal epithelia [59,79,80], and activates intracellular signals regulating cell survival, proliferation, and DNA stability, through key oncogenic molecules, like NF-κB [81] and p53 [82]. Furthermore, p75NTR regulates the expression of pluripotency transcription factors, including Sox2, Nanog, and MYC, promotes CSCs self-renewal in breast cancer, and facilitates symmetric divisions in slow-proliferating or quiescent CSCs [83]. The p75NTR neutralization with a specific monoclonal antibody prevents CSCs survival signaling mediated by ERK in HNSC, and genetic loss of p75NTR in melanoma cells completely inhibits tumorigenicity in the xenograft model [84].

### Insulin and NGF signalings cross-talk in CSCs metabolism

CSCs expansion and spreading strictly depend on the energy intake by glucose uptake. The insulin/ insulin-like growth factor (IGF) pathway is well known to control glucose uptake in the peripheral organs, as well as in the nervous system. Disturbances in the insulin/IGF pathways have been linked to the pathogenesis of cancer [85]. CSCs stemness maintenance depends on the IGF-1 receptor activation in HNSCC [86]. Accordingly, metformin, the most used drug against insulin resistance in Type 2 Diabetes, has been shown to reduce CSCs reservoir in breast cancer [87]. In particular, the insulin receptor substrate 1 (IRS1)/PI3K/AKT pathway leads to Glycogen synthase kinase 3β (GSK3β) inactivation by serine 9 phosphorylation, thus preventing Snail and Slug proteasome targeting, and activating the NF-kB transcription factor, which in turn increases Snail and Zinc finger E-box-binding homeobox (ZEB) 1 transcription for EMT (reviewed in [88]). Thus, the insulin/IGF pathway induces EMT transcription factors by cross-talking with the signaling platforms implicated in cell proliferation and pluripotency. NGF has been demonstrated to control insulin signaling and improve insulin resistance by promoting glucose uptake in degenerating neurons [89]. Noteworthy, the insulin receptor (IR) signaling has been demonstrated to be transactivated by the NGF receptor TrkA via the IR or the IRS1 in neurons and in the pheochromocytoma cell line PC12 [89,90], and by oncogenic fusion protein Trk-T1 from thyroid carcinoma triggering the IRS-Growth factor receptor-bound protein *2* (IRS-Grb2) complex [91]. Of note, Grb2 and Shc signalings are sufficient *per se* to induce transformation of fibroblasts [92] and intestinal epithelial cells [93], suggesting Grb-Shc as a crucial molecular hub for common downstream pro-oncogenic pathway. These findings pinpoint a role for the NGF-TrkA/Shc and insulin/IR-IRS axes and reciprocal interplay in the regulation of CSCs glucose metabolism and tumor expansion, highlighting a novel target for precision medicine.

### Neurotrophins contribution to CSCs Epithelial-mesenchymal transition

Neurotrophins have been implicated in EMT, a process of genetic reprogramming and morphological shift from elongated epithelial cells to migrating mesenchymal-like cells, with further conversion to CSCs, leading to CSCs enrichment in the tumor microenvironment (TME). Three different transcription factors protein families, the Snail (including Snail and Slug), ZEB (ZEB1, ZEB2), and basic helix–loop–helix (including TWIST1, TWIST2, and TCF3) induce the EMT program by chromatin rearrangement and/or promoter regulation [8]. As a result, proteins of epithelial origin, like E-cadherin, are downregulated and N-cadherin, fibronectin, and vimentin are upregulated to facilitate cell motility and autonomy from niche signals [8,94]. Other EMT transcription factors are common regulator of both CSCs proliferation and EMT commitment, like CD44, Sox2, Sox9, Nanog [8].

In breast cancer, NGF/p75NTR affects epithelial markers like keratin 18, keratin 19 and E-cadherin, while promoting mesenchymal markers, like SLUG, to sustain CSCs migratory behavior [37]. NGF stimulates the expression of SNAIL1, SNAIL2 and TWIST1 in breast cancer cells [68]. Indeed, p75NTR knock-out induces loss of stemness markers, like Sox2 and Sox10, and reconverts spindle-shaped melanoma cells in epithelial-like cells [84]. The p75NTR appears to be involved in EMT phenotypic acquisition and invasiveness. The p75NTR is developmentally expressed in the nervous system and in the neural crest, where it guides fine migration of neural crest cells to form the neural tube. Interestingly enough, the same p75NTR/sortilin signaling system is involved in neural cell migration and cancer metastasis in several tumor types [95].

BDNF/TrkB signaling through AKT and MAPK downstream effectors stimulates the expression of the TWIST-SNAIL axis in rat kidney epithelial cells inducing EMT and CSCs spreading [96]. BDNF through p75NTR activates PI3K/AKT pathway interfering with the RhoA pathway, causing cytoskeletal rearrangement, and promoting CSCs invasiveness in lung cancer, ESCC [97], and head and neck cancer [98]. siRNA silencing or Trk inhibitors prevented BDNF-mediated expression of EMT transcription factors and affected melanoma sphere-forming potential [99].

### Angiogenesis in tumor immune evasion

Sufficient blood supply to the tumor comes from neoformation of vessels through endothelial cells angiogenesis and vasculogenesis, as well as by vasculogenic mimicry. CSCs have been observed to be involved in both mechanisms [100]. Interestingly, angiogenic factors overexpressed in many cancers, like Vascular Endothelial Growth Factor (VEGF), Cyclooxygenase 2 (COX-2), and Prostaglandin E2 (PGE2) have an immunosuppressive action, supporting tumor immune evasion. VEGF also inhibits T cell development and is associated with high metastatic potential and poor prognosis in ovarian cancer. COX-2 is implicated in the production of immunosuppressive prostaglandins, like PGE2 and Prostaglandin D2 (PGD2). PGE2 downregulates TNF-α, inhibits T and B cells proliferation, and NK-mediated tumor clearance, while PGD2 favors T helper 2 cells activation at the expenses of tumor eradicating T helper 1 immune response. Also, proangiogenic microRNA (miRNA), like miR-126 sustain cancer metabolism through the IRS1-mediated pathway of glucose uptake [101]. Overall, angiogenesis favors tumor growth by a dual mechanism: on one side facilitating blood supply, on the other side through active suppression of innate and adaptive immune responses. Neurotrophins released by stromal or immune cells in the TME are known to exert a strong proangiogenic effect in *in vitro* models and *in vivo* [102–104]. In particular, NGF is known to induce angiogenesis in endothelial cells of several origins through VEGF expression. In line, NGF promotes angiogenesis by TrkA-mediated activation of PI3K and matrix metalloproteinase 2 (MMP2) in breast [105], ovarian [106], hepatocellular [107] cancers. Opposite, proNGF has a net anti-angiogenic effect, increasing angiostatin and thrombospondin-1, and decreasing angiopoietin and angiopoietin-like 1.

### Neurotrophins in cholinergic nerve-cancer interplay

Cancer cells growth around nerve terminals and subsequent neural invasion is a process known as perineural invasion (PNI). The presence of nerve endings within the tumors has been described for GC, colon, prostate, breast, pancreatic, bladder, eye cancers [108–112] (reviewed in [113]). Also, neoneurogenesis with the *ex novo* formation of axons within the tumor has been observed in prostate cancer [114]. Opposite, denervation is known to positively impact on cancer dissemination and patient’s outcome since 1940 in both human and animal studies [115–117]. PNI results from a crosstalk between cancer cells releasing neurotrophins and neuropeptides, and nerve ends expressing TrkA and p75NTR receptors. CSCs respond to NGF by TrkA-mediated autocrine proliferation [118,119] and neurotransmitter release leading to axonal growth around the tumors, tumor expansion and long distance CSCs spreading through the nerves (reviewed in [113]). The neurotrophin family of Trk receptors is well known to support neuron survival and axonal growth in the nervous system, during development and after injury. Similarly to Schwann cells induced axonal regeneration upon nerve injury, NGF, BDNF, NT3 or IGF-II produced by cancer cells sustain cancer growth and PNI through the expression of neurites chemoattractant and guidance molecules associated with poor prognostic outcome (netrin, semaphorins, ephrins, and Slits) [120,121]. NGF, in its precursor or mature form, has been strongly implicated in PNI and neoneurogenesis in prostate cancer [62,122]. Cathecolaminergic, dopaminergic, serotoninergic, glutammatergic, gabaergic, and cholinergic tumor infiltrating nerves have been described so far [123]. In particular, cholinergic nerves within the stem cell-niche help regulate stem cell dynamics in both normal and neoplastic condition [124–126] (reviewed in [40]).

Acetylcholine (Ach) is one of the most important neurotransmitters targeted by cancer pharmacotherapy and its metabolism is dependent upon NGF supply [40]. Nicotine has been indicated as a key driver of tumor cells growth in mesothelioma and colon cancer, and of proliferation, cell migration, and angiogenesis in GC [127]. Nicotine and nicotinic agonists, like 4-(methylnitrosoamino)-1-(3-pyridyl)-1-butanone (NNK), lead to human small cell lung cancer (SCLC) proliferation, while the Ach functional antagonist isoproterenol induced growth of lung adenocarcinoma cells [128]. Cholinergic stimulation through muscarinic receptors induces neurotrophic molecules expression [129–131]. Among neurotrophins, NGF was specifically upregulated (20 times higher) by the cholinergic agonist carbachol in GC mouse models, as well as in human GC [40]. A cholinergic/NGF interplay has been reported in GC, with tuft cells-derived Ach inducing NGF release by CSCs expressing the muscarinic receptor 3 (M3R). In turn, the NGF released in the TME promotes nerve growth and further Ach release in a feedback loop sustaining tumor growth and metastasis. In a similar manner, PNI and lymph node spreading is sustained by a catecholamine/NGF axis in PDAC [132]. On the other hand, M3R antagonists inhibited cell growth of non-small cell lung carcinoma (NSCLC) both *in vitro* and *in vivo* [133]. Indeed, the increase of cholinergic parasympathetic fibers by neurogenesis and axonogenesis in prostate cancer xenografts model correlates with metastasis [134]. Similarly, proNGF favors tumor infiltration and affects patient’s survival in human prostate cancer [62].

Taken together, these findings strongly pinpoint the neurotrophin/Trk signaling as messengers between nerves and cancer cells, driving peritumoral innervation and consequent tumor growth and dissemination.

### Neurotrophins and exosomes-driven tumorigenesis

Tumors release extracellular vesicles, called exosomes, regulating the TME and promoting disease progression by induction of tumor tolerance and spreading, and axonogenesis (reviewed in [135]). Exosomes are 30–150 nm vesicles expressing marker proteins, such as CD63, Alix, Tsg, CD9, and CD81, and delivering cargo of intercellular messenger proteins, mRNA, non-coding RNA like long non-coding RNA (lncRNA), miRNA, DNA, and lipids [136]. Exosomes facilitate intratumoral axon growth by releasing axonal guidance molecule, like EphrinB1 [136]. Indeed, tumors compromised in exosome release are less innervated than controls and pharmacological blockade of exosome release by the multi-vesicular body (MVB) inhibitor GW4869 is effective in diminishing tumoral innervations by β-III tubulin positive “nerve twigs” [136]. Opposite, exosomes from “non-metastatic” melanoma are able to trigger NK and TRAIL-driven macrophage clearance of tumor cells, reinforcing immune surveillance at the pre-metastatic niche and exerting a pro-apoptotic effect on lung carcinoma cells [137]. Of note, neuronal exosomes are enriched with p75NTR [138], and prostate cancer exosomes carry tyrosin kinases, such as Src tyrosine kinase and IGF-1R, promoting tumor expansion and angiogenesis [139,140]. Furthermore, the BDNF receptor TrkB transfers glioblastoma aggressiveness to recipient cancer cells [141]. CSCs exosomes from a CSCs clonal line transfer SLUG and Sox2 and induce EMT through lnc-Regulator of Reprogramming (lnc-ROR) [142]. Plus, exosomal transfer of miRNA-142 from bone marrow-derived mesenchymal stem/stromal cells (BM-MSCs) stimulates colon CSCs proliferation by NUMB-mediated activation of Notch signaling [143]. Thus, active signaling molecules are transported by cancer exosomes contributing to CSCs communication with the TME and modulating cancer outgrowth metastasis and immune cells evasion. Additionally, CSCs exosomes are potential nano-carriers for drugs and vaccines, and their cargos provide potential biomarkers for early diagnosis and improved prognosis in cancer.

## Evidence of neurotrophins actions in tumor immune surveillance

Nowadays, a deeper comprehension of neurotrophins involvement in cancer immune surveillance is of foremost relevance. In fact, neurotrophins and their receptors are key molecules in survival and functions of cells of both the innate and adaptive immune system. NGF is produced in an autocrine manner by cells of the immune system, such as B and T cells, monocytes/macrophages, eosinophils, granulocytes, and mast cells [144–146]. NGF is a growth and survival factor also for B cells [147]. During inflammation, NGF synthesis is induced by inflammatory cytokines (IL-1β, TNF-α, IL-6) in different cell types. Both NGF receptors TrkA and p75NTR are expressed by immune cells, the first being anti-apoptotic and survival stimulating [148,149], the latter transmitting pro-apoptotic signals [144].

Nonetheless, it is not clear whether and through which mechanisms neurotrophins, and especially NGF, may eventually be implicated in the modulation and refinement of tumor editing/escape.

Indeed, the most relevant known effects of NGF and its receptors on key cellular substrates and molecular mechanisms of the tumor immune surveillance are reported here below (Table 1).

**Table 1:** Main findings on the role of neurotrophin NGF and its receptors TrkA and p75NTR in tumor surveillance by innate and adaptive immune cells.

**Neurotrophins and receptors**		**Target immune cells**		**Function/effect**	**References**
NGF	NK cells		negative influence on NK cell degranulation		[161]
	CD4^+^ T-cells		regulation of immune response		[153,156]
	CD8^+^ T cells		regulation of immune response		[153,156]
TrkA	NK cells		anti-tumoral effect		[161]
	Monocytes		anti-inflammatory effect (by blocking NF-kB proinflammatory pathways and inducing anti-inflammatory cytokines)		[169]
	CD4^+^ T cells		activation and NGF synthesis and release		[155]
p75NTR	Dendritic cells		activation and induction of TLR4 expression		[162]
	γδ T cells		regulation of γδ T cells activation in autoimmune inflammation		[169,170]
	CD8^+^ T cells		activation by TCR stimulation		[178]
	Monocytes/Macrophages		increased calcium spiking, phagocytosis, TGF-β secretion, and reduced M2 marker CD206 by NGF binding; proNGF increased migration through podosome formation and neurotoxin secretion by proNGF binding		[173]

### Innate immunity

### NK cells

NK cells show the ability to selectively kill human colon-derived CSCs, melanoma, and glioblastoma without any pharmacological pretreatment *in vitro* [150–153]. Mouse resting NK cells express physiological level of TrkA, which is dramatically upregulated upon activation of NK cells by IL-2 [154], suggesting an immune stimulatory and anti-tumoral effect of the NGF-TrkA pathway mediated by NK cells. A NK subpopulation expressing the neural adhesion molecule (N-CAM) and driving local response to IL-2 has been proposed to be crucial for immune surveillance of neoplasia.

### Dendritic cells

The activation of human DCs requires signaling driven by Toll-like Receptor 4 (TLR4). Noteworthy, NGF has been shown to promote TLR4 expression following Lipopolysaccharide (LPS) treatment by p75NTR-dependent activation of p38 MAPK and NF-κB pathways [155]. On the other hand, neurotrophins have been shown to promote release of several growth factors reducing the effectiveness of DCs for immune surveillance of tumors, like IL-6, IL-10, macrophage colony-stimulating factor (MCSF), VEGF, and PGE2 [156].

### Patrolling monocytes

Non-classical patrolling monocytes are known to be early interactors of metastasizing tumor cells and to promote NK cells recruitment and activation [157,158], thus contributing to cancer immune surveillance and representing putative targets for cancer immunotherapy. Human monocytes have been reported to respond to an immunogenic stimulus by increasing TrkA mRNA expression [159].

### γδ T cells

Unlike the αβ T cells commonly used in Chimeric Antigen Receptor Therapy (CAR-T), γδ T cells play a role in the innate immune response, which constitutes the first, faster line of defense of the immune system. The γδ T cells are major players in the “lymphoid stress surveillance,” i.e., by early activation following infections or non-microbial stress. This peculiar type of T cells does not require clonal expansion or differentiation, as it occurs in the prototypic innate immunity [160]. Tumor-infiltrating γδ T cells are an important subset of “unconventional” T lymphocytes as they have the ability to recognize a broad range of antigens without the presence of MHC. The γδ T cells have been demonstrated to be the most favorable prognostic immune population among many cancer types, in agreement with their killing capacity against leukaemia, neuroblastoma, and carcinoma. By applying CIBERSORT, a computational method for inferring leukocyte representation in bulk tumors, the most favorable predictor gene is CD161, a surface molecule associated to tumor infiltration by γδ T cells [161]. They produce IL-17, IFN-γ and TNF-α, leading to DCs maturation, and prime CD4^+^ and CD8^+^ T cells.

The p75NTR has a regulatory effect on immune cells activation during autoimmunity [162] and, noteworthy, it is involved in control of γδ T cells activation in inflammation [163].

### Innate lymphoid cells

Innate lymphoid cells (ILCs) represent a constitutive patrolling immune unit aimed at tissue homeostasis maintenance at the mucosal barriers. Three main distinct ILCs subpopulations have been described, based on their phenotype and functions. ILCs type 1 produce IFN-γ; ILCs type 2 secrete IL-5 and IL-13; ILCs type 3 release IL-17 and/or IL-22 [164,165]. ILCs play an essential role in tissue inflammation and remodeling, and in cancer as well [166,167].

Of note, the localization of ILCs at cholinergic, adrenergic, and nociceptor sensory neuronal terminals in several tissues led the hypothesis of a key role in neuro‐immune interactions in normal tissue physiology and in the perineural cancer niche. In line with this, ILCs express receptors for neurotransmitters and neuropeptides, like β2‐adrenergic receptor (β2‐AR), muscarinic cholinergic receptor, vasoactive intestinal peptide receptor (VPAC1/2), and calcitonin receptor‐like for CGRP [168].

### Acquired immunity

The γδ T cells, NK cells, and Cytotoxic T Lymphocytes (CTLs) are important players in the eradication of CSCs. However, only effector cells of the adaptive immunity system may specifically recognize CSCs.

### Disabled antigen presenting cells

The presence of MHC proteins at the CSCs membrane surface is crucial for T cells-dependent anti-cancer immunity [25]. Lack or *de novo* mutation in the antigen presentation machinery may result in immune escape of malignant cells. Indeed, MHC-I down-regulation has been reported in around 40% of common solid malignancies (melanoma, lung, breast, renal, prostate, and bladder cancers). Accordingly, alterations in MHC expression have been found to correlate with the clinical outcome in cancer patients. Although CD8^+^ CTLs are deficitary in targeting CSCs with low MHC-I expression, recent *in vitro* findings suggest that human γδ T cells are able to target CSCs upon CSCs sensitization by bisphosphonate zoledronate [169]. Noteworthy, TrkA-positive neuroblastoma cells have higher amount of MHC-I complexes and a less malignant phenotype, pin-pointing the role of TrkA in neuroblastoma spontaneous regression [170].

While M1 macrophages are implicated in eradication of the foreign cell during acute phase inflammation, their polarization switch to an anti-inflammatory tumor-permissive phenotype (M2) allowing metastatic spreading is typical of TAMs and it is associated with poor prognosis. Noteworthy, cultured human macro-phages express both TrkA and p75NTR receptors and differently respond to NGF and proNGF by augmenting calcium spiking, phagocytosis, TGF-β secretion and by a slight reduction of the M2 marker CD206 in the first case, while increasing migration by podosome formation and neurotoxin secretion, in the latter [171].

### Cell dysfunction/tolerance

Main effectors of cellular acquired immunity against cancer are CD8^+^ and CD4^+^ T cells [172]. Tumor infiltration by CD8^+^ T cells is associated with prolonged patient survival [172,173]. Malignant cells elimination by CD8^+^ CTLs has been considered for decades as a master regulator of anti-tumor immunity, confining CD4^+^ T cells to a supportive action.

Nonetheless, CD4^+^ T cells have been recently found to exert a broad range of action in tumor rejection. They show cytotoxic effects on tumor cells, upregulate MHC molecules expression, are anti-angiogenic, and are able to promote tumor dormancy. By partnering with NK cells, CD4^+^ T cells maximize their ability to eliminate tumors resistant to CD8-mediated rejection, even in case of MHC-II negative tumors [174]. Noteworthy, CD4^+^ T cells are critical for expansion, trafficking and functioning of cytotoxic CD8^+^ and memory T cells, an effect known as “CD4^+^ T-cell help” fostering tumor destruction through cytokine signaling, especially IFN-γ and TNF-α [175]. Of note, CD4^+^ T-cell line 9/6 express the NGF receptor TrkA after TCR-mediated activation by the antigens and/or the antigen presenting cells (APC [148];). Activated CD4^+^ T-cell clones not only express TrkA but they also produce NGF, further pinpointing the NGF/TrkA system in an autocrine/paracrine loop modulating the maturation and activity of T cells [146,149]. As for the common neurotrophins receptor, p75NTR is implicated in antigen-driven T cell responses *in vivo* and contribute to T cell activation upon stimulation [176]. p75NTR genetic ablation *in vivo* leads to an hypoproliferative response to TCR agonists, decreased expression of the activation markers CD25 and CD69, of IL-2, and IFN-γ [176]. Moreover, activation threshold CD8^+^ CTLs upon TCR stimulation depends on p75NTR and increases following p75NTR deletion [176].

However, CD8^+^ CTLs are often unable to eradicate the tumor because of inhibition by other, immunosuppressive cells in the TME, such as Tregs. Tregs are a specialized subpopulation of CD4^+^CD25^+^ T cells producing IL-17, also called T helper 17 cells. Their induction of self-tolerance and inhibition of both natural and induced anti-tumor immunity are considered key events in cancer immune evasion. Consequently, there is considerable interest in therapeutic Tregs blockade to treat cancer [177]. Noteworthy, NGF anti-inflammatory effect through IL-6 and IL-10 down-regulate Tregs homeostatic responses and reduces IL-17 level in airway allergy [178]. It would be interesting to find out whether a similar mechanism under the control of neurotrophins takes place to counteract Tregs immune tolerance in cancer.

### Neurotrophins control of oncogenic inflammation

NGF increase at the sites of inflammation and in systemic circulation is a common event in different inflammatory and autoimmune diseases, and in cancer. Inflammatory cytokines, like IL-1β, IL-6, and TNF-α stimulate production of NGF in several cell types. In turn, NGF induces IL-10 and IL-1 receptor antagonist via the PI3K/AKT pathway and facilitates TLR4-mediated inhibition of NF-kB, leading to resolution of the inflammation [179]. The findings reported above on the role of neurotrophin signaling in the activation of immune surveillance mechanisms could be in apparent contradiction with their role in oncogenic inflammation and their classic anti-inflammatory effect reported in the Central Nervous System *in vitro* and *in vivo* [179–181]. On the contrary, NGF exerts a dual role by activating immune responses following acute insult, while concomitantly avoiding tumor growth sustained by chronic inflammation via a timely resolution of the immune response.

## Conclusions

**Figure 2:**
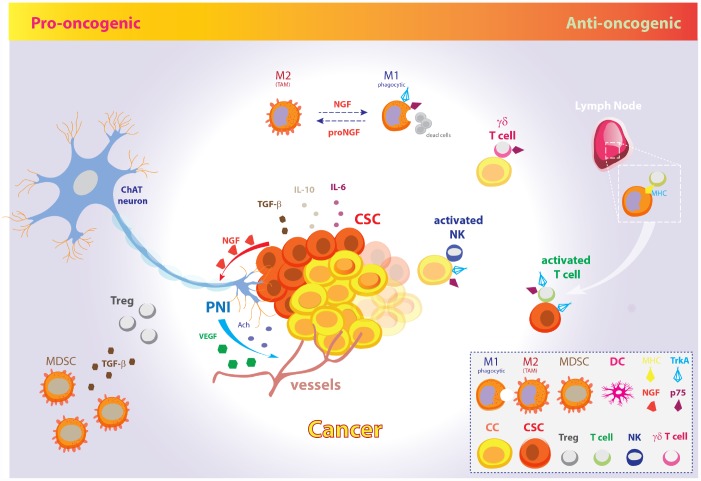
**The pro-oncogenic and anti-oncogenic effects of the NGF signaling pathway in CSC metabolism and EMT.** Schematic model illustrating the opposite pro-oncogenic (left) and anti-oncogenic (right) actions of NGF signaling pathway in the control of CSCs growth and cancer evasion from the host immune system. *Pro-oncogenic pathway*. CSCs promote tumor growth, perineural invasion, CSCs proliferation and spreading through vessels and nerves by NGF release. In fact, tumor-released NGF attracts cholinergic endings and promotes cancer expansion and neoangiogenesis through neuronal-derived Ach and VEGF. Further, CSCs inhibit the host immune response and facilitate metastatic spreading through IL-10, IL-6, and TGF-β. Excess amount of proNGF stimulates macrophages polarization toward the M2 phenotype, giving rise to TAMs, which are unable to phagocytize cancer cells. Moreover, MDSCs induce Tregs expansion by TGF-β release and contribute to dismount the T-cells mediated immune response. *Anti-oncogenic pathway*. On the other hand, increasing evidences pinpoint a role for NGF pathway in promoting tumor surveillance by both natural and adaptive immune cells. The NGF-TrkA signaling system induces phagocytic M1 macrophages, thus resolving cancerogenic inflammation. Moreover, NGF receptors allow membrane exposure of activatory NK receptors. The p75-expressing γδ T cells are phagocytic T cells of the so-called “lymphoid stress surveillance” system. NGF-TrkA promotes MHC-I and MHC-II expression by cancer cells and CSCs, and allow recruitment of IL-2 activated T cells in lymph- , promoting the tumor mass eradication. The illustration includes images modified from “freevector.com”, distributed under the Creative Commons Attribution 4.0 license (CC BY 4.0).

Taken together, the experimental findings here reported suggest opposite pro-oncogenic and anti-oncogenic actions of the NGF signaling pathway in the control of CSCs growth and cancer evasion from the host immune system (Figure 2). NGF/TrkA signaling and cholinergic innervation of the tumor niche take central stage in tumor initiation and progression, as well as metastatic spreading. In line with this, the targeting of neurotrophic growth factors in cancer has been suggested to halt tumor progression through direct CSCs targeting, to achieve control of nerve infiltration and angiogenesis, and minimize cancer pain [182–184]. Accordingly, selective and *in situ* treatment with potent Trk kinase inhibitors, clinical development of drugs targeting *NTRK* genetic rearrangement combined with canonical cancer therapy, and/or novel encouraging CAR-T immunotherapy are all promiseful neurotrophin-based strategies to improve cancer patients outcome.

Nonetheless, increasing evidences pinpoint the involvement of neurotrophins, and specially NGF, in tumor immune surveillance through cytokines-driven modulation of the innate and acquired immune system cells. In particular, given the Tregs and PD1 control by p75NTR and NGF, NGF pathway inhibition would result in immune tolerance induction, thus challenging the use tout-court of TrkA inhibitors to dampen tumor growth. Moreover, the activation of the neurotrophic pathway is fundamental in order to downregulate oncogenic inflammation during self-promoted tumor growth. To overcome this dichotomy, fine understanding of the molecular targets and cellular substrates of NGF/TrkA involved in tumor growth on one side and immune surveillance on the other side is demanding nowadays in order to design selective and coordinated therapies against tumor characterized by uncontrolled overactivation of the NGF pathway as oncogenic driver.
